# Non-alcoholic Fatty Liver Disease Induced by Perinatal Exposure to Bisphenol a Is Associated With Activated mTOR and TLR4/NF-κB Signaling Pathways in Offspring Rats

**DOI:** 10.3389/fendo.2019.00620

**Published:** 2019-09-10

**Authors:** Ren Lin, Dan Wu, Feng-Juan Wu, Yuan Meng, Jin-Heng Zhang, Xiao-Gang Wang, Li-Hong Jia

**Affiliations:** Department of Child and Adolescent Health, School of Public Health, China Medical University, Shenyang, China

**Keywords:** bisphenol A, non-alcoholic fatty liver disease, mTOR, autophagy, inflammatory response

## Abstract

Accumulating evidence suggests a role of bisphenol A (BPA) in non-alcoholic fatty liver disease (NAFLD), and its mechanism may be related to the up-regulation of lipogenic genes, but the mechanism of BPA induced lipogenic gene expression remains unknown. The aim of this study was to investigate the effects of perinatal exposure to BPA on NAFLD and its mechanisms. Pregnant Sprague-Dawley rats had access to drinking water containing 1 or 10 μg/ml BPA from gestational day 6 to post-natal day 21. For 5 weeks after weaning, offspring drank normal water without BPA. Body weight, lipid profile and the expression of genes or proteins involved in mTOR mediated lipid metabolism and autophagy, as well as inflammatory response were investigated in the 8-wk-old offspring of different genders. The results showed that body weight was increased only in females, however, males, and females from dams treated with BPA had significantly excess visceral adipose tissue, which was consistent with adipocyte hypertrophy. Elevated TG levels and up-regulation of lipogenic genes or proteins in liver, such as sterol regulatory element binding protein 1 (SREBP1), acetyl-CoA carboxylase 1 (ACC1), and fatty acid synthase (FAS) were consistent with increased liver lipid droplets in offspring exposed to BPA. Compared with controls, the protein levels of InsR, p-IRS-1, IRS-1, TSC1, and TSC2 were decreased, p-PI3K, p-Akt (S473), p-Akt (T308), p-mTOR, and mTOR were increased, and the impaired autophagic degradation was evidenced by increased protein levels of p62, although the levels of p-ULK1, Beclin1, and LC3B proteins were increased in liver of BPA-exposed offspring. The levels of TLR4 and NF-κB proteins were also significantly increased, and ERα protein was significantly decreased in BPA-exposed offspring. Our findings indicate that perinatal exposure to BPA causes the development of NAFLD in both female and male offspring, which is associated with up-regulation of lipogenic genes, dysregulated autophagy and activated inflammatory response involving the PI3K/Akt/mTOR and TLR4/NF-κB pathways.

## Introduction

Non-alcoholic fatty liver disease (NAFLD) is a clinicopathologic syndrome parallel to the obesity epidemic, characterized by diffuse hepatic macrovesicular fat due to the absence of excess alcohol consumption and other well-defined liver damage factors, and may lead to severe fibrosis and cirrhosis (1). The global prevalence of NAFLD is 25.24% with the highest prevalence in the Middle East and South America and the lowest in Africa (2). In recent years, the prevalence of NAFLD in Chinese population has been rising rapidly, and the disease is becoming younger. A study showed that the prevalence of NAFLD was 35.47% in 2006, and increased to 46.46% in 2014 in the same population after follow-up for 8 years in China (3). Genetic factors, bad dietary habits, and sedentary lifestyle may promote the development of NAFLD (2). Recently, the association between bisphenol A (BPA) and NAFLD has attracted attention, because it increases the risk of obesity and related diseases (4).

BPA is one of the highest productive endocrine disrupting chemicals (EDCs), which is ubiquitous in our daily life due to its wide use in manufacturing polycarbonate plastics, epoxy resins, food containers, thermal paper, and dental sealants (5). BPA can leach from these productions, especially when exposed to high temperatures and acid and alkali conditions, resulting in widespread low-dose exposure in humans due to these multiple sources and daily contact (6, 7). BPA can be detected in human urine or placenta, cord blood, amniotic fluid and breast milk, indicating that BPA has been exposed to sensitive prenatal stages (8). Accumulating evidence has demonstrated that exposure to BPA is associated with an increased risk of obesity, insulin resistance (IR), type 2 diabetes, and dyslipidemia (9, 10)—all metabolic disorder events that may induce NAFLD. In rodents, perinatal exposure to BPA leads to hepatic lipid accumulation and increased lipogenic gene expression, along with disturbances in adipokines and insulin signaling in adolescent and adult female offspring (11). A recent study reported that elevated levels of BPA in the urine are associated with NAFLD (12). The mechanism of hepatic lipid accumulation induced by BPA is related to the up-regulation of lipogenic genes, such as sterol regulatory element binding protein 1 (SREBP1) (1), but the reason for increased SREBP1 gene or protein expression induced by BPA has not been elucidated. Microarray analysis showed that BPA-induced adipogenesis involved in SREBP1, thyroid-receptor/retinoic X receptor (TR/RXR), and the mammalian target of rapamycin (mTOR) pathways by using primary human preadipocytes (13). Another recent study indicated that BPA induces persistent fat accumulation via hypomethylation of SREBP1 gene in the liver (4). Therefore, the reasons for the increase of SREBF1 induced by BPA deserve further study.

The mTOR integrates diverse environmental signals and translates these cues into appropriate cellular responses. A growing body of evidence has revealed that activation of the mTOR signaling pathway leads to the occurrence of obesity, type 2 diabetes and malignant tumors (14, 15). mTOR is a downstream target of the phosphoinositide 3-kinase (PI3K) and protein kinase B (Akt) pathways that regulate cell growth and autophagy, as well as lipid synthesis via SREBP1. mTOR also is a pivotal component of the insulin signaling pathway. Recent studies have reported that abnormal autophagy may play an important role in the pathogenesis of NAFLD (16). Acetylshikonin improved NAFLD by increasing hepatocyte autophagy through mTOR pathway (17). Likewise, Veskovic et al. indicated that the beneficial effect of betaine in methionine-choline deficient diet (MCD)-induced NAFLD is related to reduced oxidative stress, inflammation and apoptosis, and increased autophagy (18). Whether BPA-induced NAFLD is associated with autophagy has not been established.

The aim of the present study was to examine the associated between perinatal exposure to BPA with NAFLD, and its possible mechanism in offspring of different gender. Hence, we hypothesized that abnormal lipid metabolism, autophagy, and inflammatory response may be involved in the pathogenesis of NAFLD induced by perinatal exposure to BPA through mTOR and nuclear factor-κB (NF-κB) signaling pathways.

## Materials and Methods

### Animals

**Figure 1:**
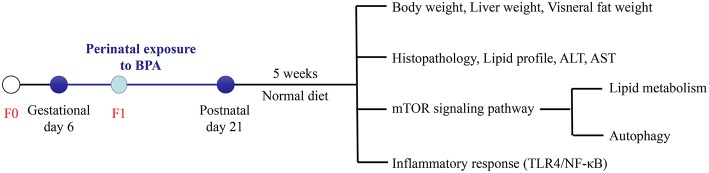
Experimental design. Pregnant rats (F0) were exposed to BPA from gestational day (GD) 6 to post-natal day (PND) 21. At 8 weeks of age, the influence of perinatal exposure to BPA on NAFLD and its possible mechanism in offspring were studied.

Sprague-Dawley (SD) rats were received from the Center for Experimental Animals at China Medical University (Shenyang, China) with a National Animal Use License number of SCXK-LN 2013-0007. All experiments and surgical procedures were approved by the Animal Use and Care Committee at China Medical University, which complies with the National Institutes of Health Guide for the Care and Use of Laboratory Animals. All efforts were made to minimize the number of animals and their suffering. Rats were housed at 24 ± 1°C temperature, 45 ± 5% humidity and a 12-h light/12-h dark cycle. Female SD rats were randomly assigned into control group (*n* = 6), 1 μg/ml BPA (LBPA) group (*n* = 6), and 10 μg/ml BPA (HBPA) group (*n* = 6) after adaptive feeding for 1 week and then mated with the normal male rats. The date on which the vaginal plug was observed as gestational day (GD) 0. To mimic the most likely route of human exposure, rats were exposed to BPA (99% pure; Sigma-Aldrich, St. Louis, MO, USA) through drinking water from GD 6 to post-natal day (PND) 21. For 5 weeks after weaning, offspring had *ad libitum* access to standard chow and drank normal water without BPA. Two female and two male offspring were chosen randomly from each litter (*n* = 12/group) when the experiment was over. Body weight (BW) of the offspring was recorded weekly. At the end of the study, offspring rats were sacrificed after overnight fasting. The body length of the offspring was measured for calculating Lee's index according to the following equation: Lee's index = body weight (g) × 10/body length (cm). Blood collected from tail were used to measure fasting blood glucose (FBG) levels with a glucometer (Sinocare, Changsha, China), and blood collected from aorta abdominalis were centrifuged to separate the serum and then frozen at −80°C for use. Liver and visceral adipose tissue (perigonadal and perirenal adipose tissue) were collected and weighed. Portions of the liver or perigonadal adipose tissue were either frozen at −80°C for next analysis or fixed in tissue fixative solution for further histopathological analysis. A schematic representation of the protocol for the treatment of the animal is the following (Figure 1).

### Measurements

Liver triglyceride (TG), and serum TG, total cholesterol (T-CHO), LDL cholesterol (LDL-C), HDL cholesterol (HDL-C), aspartate aminotransferase (AST), and alanine aminotransferase (ALT) were measured by using commercial assay kits (Nanjing Jiancheng Bioengineering, Nanjing, China). Serum insulin, interleukin-6 (IL-6) and tumor necrosis factor-α (TNF-α) were measured by ELISA assay kits (Shanghai Enzyme-linked Biotechnology, Shanghai, China). All assays were carried out according to the manufacturer's instructions. Homeostasis model assessment of insulin resistance (HOMA-IR), which indicates the degree of insulin resistance, was calculated according to the following equation: HOMA-IR = FBG (mmol/l) × fasting insulin (mIU/l)/22.5 (19).

### Histology

Rat liver and perigonadal adipose tissues were fixed in 4% paraformaldehyde and embedded in paraffin wax after dehydration. Embedded tissues were cut into 5 μm sections and stained with hematoxylin and eosin (H&E). Frozen liver tissues were embedded in the OCT compounds and cryosections (8–10 μm thick) were prepared. The sections were then stained with Oil Red O solution. All sections were scanned with a scanner (Aperio ScanScope CS^2^, Leica Biosytems Imaging, Inc., USA) and then quantified using Image-Pro Plus software (version 6.0; Media Cybernetics, Inc., Rockville, MD, USA).

### Real-Time PCR

Total RNA was isolated from liver using the TRIzol Reagent (Invitrogen, Carlsbad, CA, USA) and then reverse transcribed into cDNA with the PrimeScript RT reagent Kit (DRR047A, Takara, Japan). The cDNA was served as templates for real-time PCR amplification by using the SYBR® Premix Ex Taq™ II (Takara, Japan) and QuantStudio 6 Flex Real-Time PCR System (Life Technologies). Relative mRNA expression levels were determined by the 2^−ΔΔCt^ method with GAPDH as an internal reference, and presented as fold change vs. control sample. The primer sequences are given in Table 1.

**Table 1:** Primer sequences for quantitative real-time PCR.

**Gene name**	**Forward primer sequence**	**Reverse primer sequence**
*InsR*	5′-TTCATTCAGGAAGACCTTCGA-3′	5′-AGGCCAGAGATGACAAGTGAC-3′
*IRS-1*	5′-AGAGTGGTGGAGTTGAGTTG-3′	5′-GGTGTAACAGAAGCAGAAGC-3′
*PI3K*	5′-GAAGGCAACGAGAAGGA-3′	5′-CGTCAGCCACATCAAGTA-3′
*Akt*	5′-ACCTCTGAGACCGACACCAG-3′	5′-AGGAGAACTGGGGAAAGTGC-3′
*mTOR*	5′-GACAACAGCCAGGGCCGCAT-3′	5′-ACGCTGCCTTTCTCGACGGC-3′
*SREBP1*	5′-GTGGTCTTCCAGAGGCTGAG-3′	5′-GGGTGAGAGCCTTGAGACAG-3′
*ACC1*	5′-AGGAAGATGGTGTCCCGCTCTG-3′	5′-GGGGAGATGTGCTGGGTCAT-3′
*FAS*	5′-CCAAGCAGGCACACACAATG-3′	5′-GAGTGAGGCCGGGTTGATAC-3′
*ATGL*	5′-AGACTGTCTGAGCAGGTGGA-3′	5′-AGTAGCTGACGCTGGCATTC-3′
*HSL*	5′-CTCCCAAAGTAAGAGGCACAGA-3′	5′-CATGATGGCACCTCCCTTTG-3′
*GAPDH*	5′-GCAAGAGAGAGGCCCTCAG-3′	5′-TGTGAGGGAGATGCTCAGTG-3′

*InsR, insulin receptor; IRS-1, insulin receptor substrate-1; PI3K, phosphoinositide 3-kinase; Akt, protein kinase B; mTOR, mammalian target of rapamycin; SREBP1, sterol regulatory element binding protein 1; ACC1, acetyl-CoA carboxylase 1; FAS, fatty acid synthase; ATGL, adipose triglyceride lipase; HSL, hormone sensitive lipase*.

### Western Blotting

Total proteins were extracted from livers of rat offspring using a commercial kit. Protein concentrations were detected with a BCA protein assay kit (Beijing Dingguo Changsheng Biotechnology, Beijing, China). Protein samples were then mixed with loading buffer and boiled for 5 min. A total of 50 μg protein (i.e., 10 μg aliquots) was subjected to SDS-PAGE (SDS-polyacrylamide gel electrophoresis). Following electrophoresis, proteins were transferred to a polyvinylidene difluoride (PVDF) (Millipore, Bedford, MA, USA) membrane electronically. The membrane was blocked with 5% BSA for 1 h at room temperature and incubated with a primary antibody overnight at 4°C. The primary antibodies included: rabbit anti-fatty acid synthase (FAS), rabbit anti-insulin receptor (InsR), rabbit anti-insulin receptor substrate-1 (IRS-1), rabbit anti-phosphorylated (p)-IRS-1, rabbit anti-PI3K, rabbit anti-Akt, rabbit anti-p-Akt (S473), rabbit anti-p-Akt (T308), rabbit anti-tuberous sclerosis complex 1 (TSC1), rabbit anti-tuberous sclerosis complex 2 (TSC2), rabbit anti-mTOR, rabbit anti-p-mTOR, rabbit anti-p-UNC-51-like kinase 1 (ULK1), rabbit anti-light chain 3B (LC3B), rabbit anti-SQSTM1/p62, rabbit anti-Beclin-1, rabbit anti-NF-κB, rabbit anti-inhibitor of kappa B alpha (IκBα), rabbit anti-GAPDH were procured from Cell Signaling Technology (CST, MA, USA) (dilution 1:1,000); rabbit anti-p-PI3K and rabbit anti-hormone sensitive lipase (HSL) purchased from Abcam (Cambridge, MA, USA) (dilution 1:1,000); rabbit anti-SREBP1 purchased from Novus biological (Littleton, CO, USA) (dilution 1:1,000); rabbit anti-Zinc-α2-glycoprotein (ZAG), rabbit anti-toll-like receptor-4 (TLR4), and rabbit anti-estrogen receptor α (ERα) purchased from Santa Cruz Biotechnology (Santa Cruz, CA, USA) (dilution 1:200). After incubation, the membranes were washed three times in TBS containing 0.1% Tween20 (TBST), 10 min a time, and followed by incubation with horseradish peroxidase (HRP)-conjugated anti-rabbit IgG antibody (AS014, Abclonal, Wuhan, China) or HRP-conjugated anti-mouse IgG antibody (AS003, Abclonal, Wuhan, China) (dilution 1:5000) for 1 h. Specific bands were visualized by ECL detection and quantified via Image J software (Bio-Rad, Hercules, CA, USA). Relative protein expression of target band was equal to the ratio of gray value of target protein to that of internal control GAPDH.

### Statistics

All statistics were carried out using SPSS 21.0 software (SPSS Inc., Chicago, IL, USA) and all experiments were performed in at least triplicate. Data are expressed as means ± standard deviations (SD). Differences between groups were analyzed by one-way analysis of variance (ANOVA). A *p* < 0.05 was considered statistically significant.

## Results

### Perinatal Exposure to BPA Induced Lipid Metabolism Disorder

#### Effects of Perinatal Exposure to BPA on Body Weight and Lee's Index

**Figure 2:**
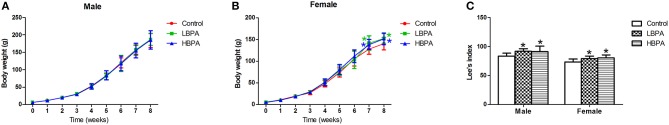
Effects of perinatal exposure to BPA on body weight and Lee's index of offspring. **(A,B)** Effects of perinatal exposure to BPA on body weight of offspring. **(C)** Perinatal exposure to BPA increased Lee's index of offspring. Data are expressed as means ± SD, *n* = 12. Statistical significance was determined by one-way ANOVA. *p < 0.05 compared to control group. LBPA, 1 μg/ml BPA; HBPA, 10 μg/ml BPA.

In this study, the body weight of each group increased with time, and there was no significant difference in body weight of LBPA or HBPA groups male offspring compared with control group, while the body weight of female offspring increased from seven7th week. Lee's index, an index to evaluate the degree of obesity in rats, was higher in offspring of LBPA and HBPA groups than those in control group (Figure 2).

#### Effects of Perinatal Exposure to BPA on Lipid Profile, Hepatic Function, and HOMA-IR

The weight of liver, perigonadal adipose and perirenal adipose was measured and the coefficient was computed. The liver coefficient of offspring in LBPA and HBPA groups was higher than that in control group, but there was no significant difference. Due to the more perigonadal adipose, the visceral adipose coefficient of LBPA and HBPA groups male offspring was higher than that of control group. For female offspring, the coefficient of perigonadal, perirenal, and visceral adipose in LBPA and HBPA groups was higher than that in control group, and the difference was statistically significant.

Compared with the control group, for male offspring, the serum levels of TG and T-CHO in LBPA group, AST in HBPA group, and ALT in LBPA and HBPA groups were higher than those in control group; for female offspring, the serum levels of TG, T-CHO, AST, and ALT were increased in LBPA and HBPA groups. And there were higher levels of LDL-C and lower levels of HDL-C in LBPA and HBPA groups offspring compared with control group. There was no significant difference in FBG level, but the insulin level was significantly higher in offspring treated with BPA compared with control group. Except for the male offspring of HBPA group, the HOMA-IR of the BPA exposed offspring groups was statistically higher than that of control group. There were significantly higher TG levels in liver of male and female offspring than that in controls (Table 2).

**Table 2:** Effects of perinatal exposure to BPA on parameters of offspring.

**Parameter**	**Male**			**Female**		
	**Control**	**LBPA**	**HBPA**	**Control**	**LBPA**	**HBPA**
Liver coefficient (%)	3.50 ± 0.23	3.71 ± 0.46	3.55 ± 0.27	3.32 ± 0.25	3.57 ± 0.38	3.42 ± 0.15
Perigonadal adipose coefficient (%)	0.40 ± 0.05	0.51 ± 0.04^*^	0.50 ± 0.06^*^	0.11 ± 0.05	0.18 ± 0.08^*^	0.15 ± 0.04^*^
Perirenal adipose coefficient (%)	0.18 ± 0.06	0.17 ± 0.04	0.18 ± 0.04	0.21 ± 0.04	0.29 ± 0.07^*^	0.26 ± 0.06^*^
Visceral adipose coefficient (%)	0.59 ± 0.09	0.68 ± 0.09^*^	0.67 ± 0.09^*^	0.32 ± 0.08	0.47 ± 0.14^*^	0.42 ± 0.10^*^
TG (mmol/l)	0.12 ± 0.03	0.21 ± 0.08^*^	0.16 ± 0.05	0.12 ± 0.04	0.18 ± 0.03^*^	0.21 ± 0.07^*^
T-CHO (mmol/l)	1.41 ± 0.15	1.68 ± 0.26^*^	1.45 ± 0.30	1.13 ± 0.12	1.48 ± 0.25^*^	1.71 ± 0.20^*^
LDL-C (mmol/l)	0.32 ± 0.08	0.57 ± 0.08^*^	0.53 ± 0.12^*^	0.24 ± 0.05	0.36 ± 0.11^*^	0.40 ± 0.09^*^
HDL-C (mmol/l)	1.23 ± 0.32	0.86 ± 0.24^*^	0.73 ± 0.12^*^	1.20 ± 0.17	0.76 ± 0.12^*^	0.88 ± 0.14^*^
AST (U/l)	10.60 ± 2.55	13.90 ± 3.44	15.12 ± 3.14^*^	12.80 ± 2.50	19.44 ± 1.18^*^	17.21 ± 2.79^*^
ALT (U/l)	10.35 ± 2.41	15.79 ± 3.84^*^	16.94 ± 2.94^*^	5.70 ± 1.24	10.35 ± 3.67^*^	8.97 ± 1.96^*^
FBG (mmol/l)	4.24 ± 0.44	4.66 ± 0.43	4.76 ± 0.55	4.11 ± 0.43	4.44 ± 0.65	4.47 ± 0.61
Insulin (mIU/l)	5.16 ± 0.64	5.87 ± 0.30^*^	5.79 ± 0.33^*^	5.81 ± 0.94	7.31 ± 1.32^*^	8.40 ± 1.49^*^
HOMA-IR	1.17 ± 0.18	1.49 ± 0.46^*^	1.23 ± 0.26	1.01 ± 0.22	1.48 ± 0.28^*^	1.63 ± 0.35^*^
Liver TG (mmol/g)	7.81 ± 0.99	10.36 ± 2.47^*^	9.94 ± 0.65^*^	8.10 ± 0.85	10.34 ± 1.14^*^	9.84 ± 1.68^*^

*Data are expressed as means ± SD, n = 12. Statistical significance was determined by one-way ANOVA*.

* *p < 0.05 compared to control group. LBPA, 1 μg/ml BPA; HBPA, 10 μg/ml BPA*.

#### Liver and Adipose Tissue Morphology

**Figure 3:**
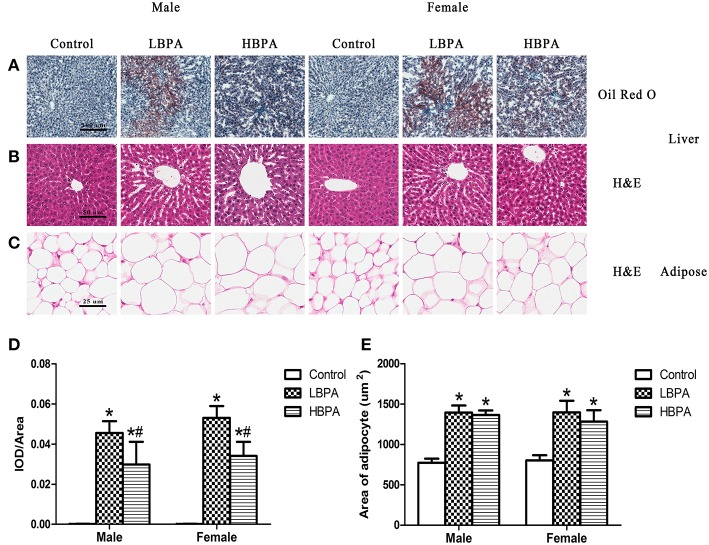
Histological examination of the liver tissue and perigonadal adipose tissue in perinatal exposure to BPA offspring. **(A)** Representative images of liver section stained with Oil Red O (100×). **(B)** Representative images of liver section stained with HE (200×). **(C)** Representative images of adipose section stained with HE (400×). **(D)** Integrated optical density (IOD)/area showing the mean value of cumulative Oil Red O optical density within the statistically effective area. **(E)** The area of adipocyte from the HE stained sections of adipose tissue. Data are expressed as means ± SD. Statistical significance was determined by one-way ANOVA. *p < 0.05 compared to control group and ^#^*p* < 0.05 compared to LBPA group. LBPA, 1 μg/ml BPA; HBPA, 10 μg/ml BPA.

Histological changes of liver were observed on H&E and Oil Red O stained sections. In lipid Oil Red O staining, red particles represent lipids. The hepatocyte lipids in BPA group were clearly stained in red, whereas those of the control group presented only weak staining, indicating that the lipid accumulation in the liver was increased in BPA group compared with control group (Figures 3A,D). No pathological changes were observed in liver of control group offspring, which showed complete hepatocyte structure and no fat droplets, denaturation or necrosis. The offspring exposed to BPA exhibited diffuse fat deposition, especially near the central vein, manifesting as microvesicular steatosis (Figure 3B). As shown in Figures 3C,E, the size and volume of adipocytes in BPA group were markedly larger than those in control group (Figures 3C,E).

#### Effects of Perinatal Exposure to BPA on the Levels of Protein or mRNA Involved in Lipid Metabolism

**Figure 4:**
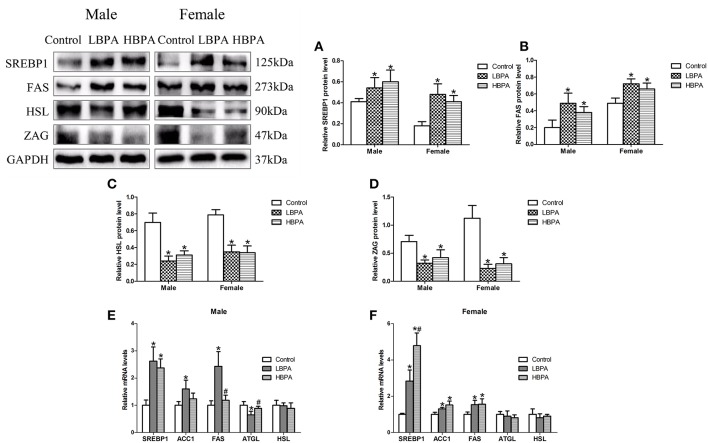
Effects of perinatal exposure to BPA on the levels of protein or mRNA involved in lipid metabolism of offspring. **(A–D)** Levels of key proteins involved in lipid metabolism. **(E,F)** mRNA expression of genes involved in lipid metabolism. Data are expressed as means ± SD. For protein, *n* = 6 rats per group, three replicates for each protein; for mRNA, *n* = 5 rats per group, three replicates for each gene; only one male offspring and one female offspring were selected per litter. Statistical significance was determined by one-way ANOVA. *p < 0.05 compared to control group and ^#^*p* < 0.05 compared to LBPA group. LBPA, 1 μg/ml BPA; HBPA, 10 μg/ml BPA.

As shown in Figure 4, SREBP1 and FAS protein levels were significantly increased, while HSL and ZAG protein levels were significantly decreased in liver of LBPA and HBPA groups compared with control group (Figures 4A–D). There were significantly higher levels of *SREBP1* mRNA in all LBPA and HBPA groups offspring, and higher levels of *ACC1* and *FAS* mRNA in LBPA group offspring and HBPA group female offspring. The levels of *ATGL* mRNA were lower in LBPA group male offspring than that in controls, however, there were no significant differences in the levels of *HSL* mRNA in all LBPA and HBPA groups offspring, and *ATGL* mRNA in LBPA and HBPA groups female offspring (Figures 4E,F).

### Perinatal Exposure to BPA Activated PI3K/Akt/mTOR Signaling Pathway

**Figure 5:**
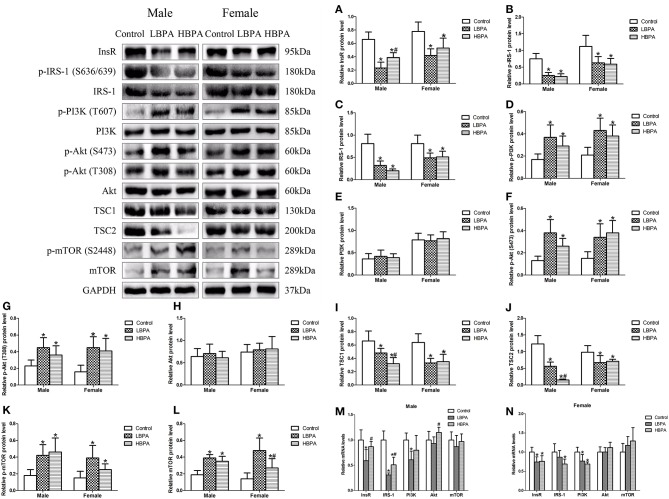
Effects of perinatal exposure to BPA on the levels of protein or mRNA involved in mTOR signaling pathway of offspring. **(A–L)** Levels of key proteins involved in mTOR signaling pathway. **(M,N)** mRNA expression of genes involved in mTOR signaling pathway. Data are expressed as means ± SD. For protein, *n* = 6 rats per group, three replicates for each protein; for mRNA, *n* = 5 rats per group, three replicates for each gene; only one male offspring and one female offspring were selected per litter. Statistical significance was determined by one-way ANOVA. *p < 0.05 compared to control group and ^#^*p* < 0.05 compared to LBPA group. LBPA, 1 μg/ml BPA; HBPA, 10 μg/ml BPA.

To determine the molecular mechanism underlying the effect of perinatal exposure to BPA on lipid metabolism, we determined the levels of protein or mRNA involved in mTOR signaling pathway in liver. As shown in Figure 5, the levels of InsR, p-IRS-1, IRS-1, TSC1, and TSC2 protein were significantly lower in LBPA and HBPA groups offspring, while p-PI3K, p-Akt (S473), p-Akt (T308), p-mTOR, and mTOR protein were significantly higher, compared with control group. The levels of PI3K and Akt protein in LBPA and HBPA groups were not significantly different from those in control group. What's more, there was no consistent dose-dependent manner on the proteins involved in mTOR signaling pathway with BPA concentration (Figures 5A–L).

Compared with control group, for males, there were lower levels of *InsR* and *PI3K* mRNA in LBPA group, and *IRS-1* mRNA in LBPA and HBPA groups; for females, there were lower levels of *InsR* and *PI3K* mRNA in LBPA and HBPA groups, and *IRS-1* mRNA in HBPA group. However, the levels of *Akt* and *mTOR* mRNA in offspring exposed to BPA did not differ from those in control group (Figures 5M,N).

### Perinatal Exposure to BPA Increased Ineffective Autophagy

**Figure 6:**
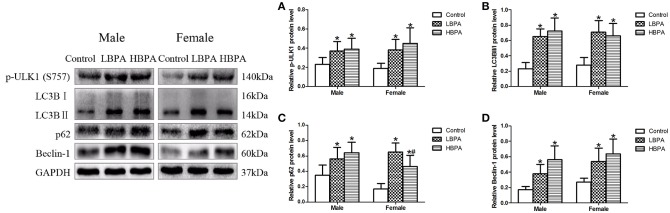
Effects of perinatal exposure to BPA on the levels of protein involved in autophagy of offspring. **(A–D)** Levels of key proteins involved in autophagy. Data are expressed as means ± SD. For protein, *n* = 6 rats per group, three replicates for each protein; only one male offspring and one female offspring were selected per litter. Statistical significance was determined by one-way ANOVA. *p < 0.05 compared to control group and ^#^*p* < 0.05 compared to LBPA group. LBPA, 1 μg/ml BPA; HBPA, 10 μg/ml BPA.

In order to further investigate the effect of BPA on autophagy, we measured the expression of autophagy markers by western blotting in liver of offspring from perinatal exposure to BPA. Our results showed that the levels of p-ULK1, LC3B II/I, p62 and Beclin-1 in LBPA and HBPA groups were significantly higher than those in control group (Figures 6A–D).

### Perinatal Exposure to BPA Activated NF-κB Signaling Pathway

**Figure 7:**
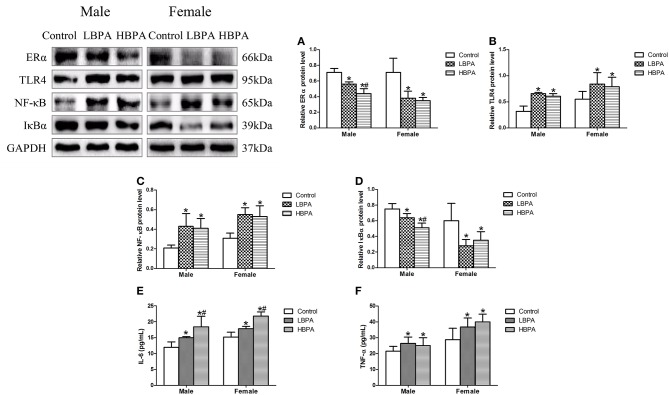
Effects of perinatal exposure to BPA on the levels of protein involved in inflammatory response and the levels of inflammatory cytokines of offspring. **(A–D)** Levels of key proteins involved in inflammatory response. **(E,F)** Levels of serum IL-6 and TNF-α. Data are expressed as means ± SD, *n* = 6; only one male offspring and one female offspring were selected per litter. Statistical significance was determined by one-way ANOVA. *p < 0.05 compared to control group and ^#^*p* < 0.05 compared to LBPA group. LBPA, 1 μg/ml BPA; HBPA, 10 μg/ml BPA.

The results showed that the levels of ERα and IκBα protein were significantly lower, and TLR4 and NF-κB protein were significantly higher in liver of LBPA and HBPA groups compared with control group. Moreover, for male offspring, the levels of ERα and IκBα protein were significantly lower in HBPA group compared with LBPA group (Figures 7A–D). In addition, the levels of serum IL-6 and TNF-α were also increased in LBPA and HBPA groups compared with control group. And HBPA resulted in a significant elevation in the level of IL-6 compared with LBPA (Figures 7E,F).

## Discussion

Epidemiological and experimental studies suggested that the prevalence of NAFLD may be associated with widespread low-level exposure to BPA (4, 20), but the mechanism of BPA action is unclear. We selected 1 and 10 μg/ml as low and high doses of BPA exposure during a critical window of development according to the relevant literature to explore the relationship between BPA and NAFLD, and its possible mechanism (21). Our major findings were that the development of NAFLD induced by perinatal exposure to BPA in female and male offspring is associated with abnormal lipid metabolism, dysregulated autophagy and activated inflammatory response involving PI3K/Akt/mTOR and TLR4/NF-κB signaling pathways.

Our results showed that perinatal exposure to BPA had an inconsistent effect on body weight in male and female offspring. Although the body weight of males did not increase, the Lee's index for evaluating the degree of obesity in rats was elevated in males and females compared with control group, which was consistent with increased visceral adipose tissue and significantly enlarged adipocytes around the genital. Consistent with our research, Somm et al. reported that perinatal exposure to 1 mg/l BPA resulted in female offspring being heavier than control group with increased perigonadal adipose tissue weight, rather than male offspring, showing that the effects of BPA exposure in a sex specific manner (21). Another study showed that perinatal exposure to 50 μg/kg BW/day BPA by oral gavage resulted in increased body weight and metabolic disease, while no adverse effects were observed in two higher doses (250 and 1250 μg/kg BW/day) (22), showing that action of BPA on multiple endpoints, including body weight and metabolic syndrome, follows a non-monotonic dose-response curve (23). So far, debate continues about the ability of BPA exposure on body weight. The contradictory results may be associated with the animal species, age and sex, dose, route and time of administration and diet etc.

There were elevated serum levels of TG and T-CHO in BPA exposed females offspring, however, the changes in HBPA group males were not been found. Elevated serum LDL-C levels and reduced HDL-C levels are strong and independent risk factors for cardiovascular diseases (CVD) (24). Our results suggest that perinatal exposure to BPA can lead to dyslipidemia and increase the risk of cardiovascular disease in male and female offspring. In recent years, the effect of BPA on lipid metabolism has attracted wide attention. Moustafa et al. showed that BPA exposure resulted in a significant increase in the serum levels of TG and T-CHO, and a significant decrease in HDL-C in mothers and female offspring (25). Our previous study also has consistent results with the above study (26). However, the mechanisms of BPA affecting glucolipid metabolism are different and insufficient *in vivo* and *in vitro* studies, involving in the peroxisome proliferator-activated receptors (PPARα, γ), the androgen receptors, ERα, β, estrogen-related receptors, TRα, β, and the pregnane X receptor (PXR) (27). Considering that there are more studies on obesity and diabetes caused by BPA, and less studies on dyslipidemia, especially on NAFLD, the relationship between BPA and lipid metabolism and its mechanism deserve further study.

Liver is a crucial organ for regulating lipid metabolism, so we carried out a comprehensive study on the liver of offspring rats after perinatal BPA exposure. There was no difference in liver coefficient, but the markers of liver damage, AST and ALT, as well as hepatic TG concentrations were significantly higher in offspring exposed to BPA than that in control group. Histopathological results showed that significantly higher degree of lipid accumulation, together with condensed hepatocytes, diffused cytoplasm were exhibited in the livers of offspring exposed to BPA. All these changes indicate that fatty liver and liver damage have occurred in BPA exposed offspring. However, offspring exposed to low doses of BPA showed more lipid accumulation than high doses from liver tissue stained with Oil Red O.

Then we explored the expression of key genes and proteins that regulate lipid metabolism in the liver. FAS and ACC1 are the key enzymes catalyzing lipogenesis, and HSL and ATGL are the key enzymes catalyzing lipolysis. SREBP1 is the most important transcription factor regulating *de novo* lipogenesis (DNL) in the liver, which can specifically regulate the expression of ACC1 and FAS (28). The imbalance between lipid input (DNL and fatty acid uptake) and output (fatty acid oxidation and TG export) processes is involved in the development of NAFLD (1). Previous evidence documented that BPA exposure stimulated SREBP1 and its downstream enzymes involved in lipogenesis, and eventually caused dyslipidemia, fat accumulation and NAFLD (29). Furthermore, our results showed that the levels of ZAG protein were lower in the liver of offspring exposed to BPA than that in controls. As a newly identified adipokine, ZAG is involved in inhibiting lipogenesis, as well as stimulating lipolysis and lipid utilization, and plays a pivotal role in the regulation of body weight and glucose and lipid metabolism (30). A study performed in humans showed that circulating ZAG levels were lower in type 2 diabetes patients than in controls, and circulating ZAG levels were correlated positively with HDL-C and adiponectin, and correlated negatively with body mass index (BMI), TG, and HOMA-IR (31).

mTOR is a highly conserved serine/threonine (Ser/Thr) kinase, which is involved in lipogenesis and autophagy (32). We examined the levels of mTOR upstream and downstream proteins in the liver to investigate the mechanism of NAFLD induced by BPA exposure. In this study, HOMA-IR was significantly higher in offspring exposed to BPA, suggesting that perinatal exposure to BPA gave rise to IR in the offspring rats. IR, as the most common pathological condition in which cells in the body become resistant to the normal functions of insulin, is associated with type 2 diabetes, hypertension, atherosclerosis and NAFLD etc. (33, 34). The binding of insulin to its receptor initiates activation of IRS-1, and activated IRS-1 phosphorylates the p85 regulatory subunit PI3k, which phosphorylates downstream Akt via its pleckstrin homology domain (35). Subsequently, Akt regulates mTOR by inhibiting TSC1/2, a negative regulator of mTOR (36). Insulin-stimulated mTOR signaling activates the expression of SREBP1 and its downstream lipogenic enzymes (ACC1 and FAS) involved in steroid and fatty acid biosynthesis, and increases lipogenesis (37, 38). Our results showed that the protein levels of InsR, p-IRS-1, IRS-1, TSC1, and TSC2 were decreased in the liver of offspring treated with BPA compared with controls, while the protein levels of p-PI3K, p-Akt (S473), p-Akt (T308), p-mTOR, and mTOR were increased. The reason for these changes may be related to the long-term activation of mTOR signaling due to excessive insulin secretion, which leads to the overexpression of mTOR and its downstream S6 kinase (S6K), and the inhibition of insulin signaling after hyperphosphorylation of IRS-1, thus causing IR. Therefore, perinatal exposure to BPA activated mTOR/SREBP1 signaling pathway, leading to up-regulation of lipogenic genes and lipid accumulation in the liver of offspring.

Autophagy is a cellular process also known as programmed cell death type 2, which can lead to a form of non-apoptotic cell death (39). The destruction of autophagy is associated with a number of metabolic disorders, including type 2 diabetes, obesity, liver damage, and NAFLD (40). A study identified a significant function of autophagy in lipid metabolism, called *lipophagy* (41). mTOR is a key negative regulator of autophagy, which phosphorylates the Ser757 site of ULK1 and contributes to inhibition of autophagy (42). During autophagy, LC3B changes dynamically, and the increase of LC3B II represents the accumulation of autophagosomes (43). p62 is a central marker of autophagic degradation, and its accumulation is considered as a hallmark of impaired autophagic degradation (44). Beclin1 is an essential molecular in the process of autophagosome formation, which can regulate the localization of other autophagy proteins to phagophore, thereby regulating the formation and maturation of autophagosomes (45).

An interesting finding in this study was that the protein levels of p-ULK1, LC3B II/I, and Beclin1 were elevated, while the protein levels of p62 were also elevated, indicating that the formation of autophagosomes was increased, but the degradation was reduced, that is, the downstream of the autophagy process was inhibited. Autophagosomes are intermediate structures in a dynamic pathway, and its number at any given time point reflects the balance between the rate of its production and degradation (44, 45). More appearance of autophagosomes does not necessarily represent enhanced autophagy, which may be due to lessened degradation, ultimately leading to ineffective autophagy. A recent study also reported that BPA exposure resulted in autophagosome accumulation in HepG2 cells and increased expression of LC3B II/I and p62 proteins, the same results were also found in the liver of male CD1 mice directly exposed to BPA in drinking water (40). Our study supports that NAFLD induced by perinatal exposure to BPA is associated with dysregulated autophagy in the liver of male and female offspring.

In general, activation of mTOR is associated with inhibition of autophagy. In our study, both p-mTOR and p-ULK1 proteins were higher in the liver of offspring exposed to BPA. Studies also showed that the expression of p-mTOR and p-ULK1 can be consistent. For example, a recent study has shown that salidroside can exert protective effects against hypoxia-induced pulmonary arterial smooth muscle cell (PASMC) proliferation and apoptosis resistance by reducing the expression of p-mTOR and p-ULK1 proteins, whereas MHY1485 (mTOR agonist) activates the expression of both p-mTOR and p-ULK1 proteins (46). Since autophagy can be regulated through a variety of signaling pathways, such as adenosine monophosphate activated protein kinase (AMPK)α1-ULK1 and AMPKα1-mTOR-ULK1 pathways, we speculate that the unanimous up-regulation of p-mTOR and p-ULK1 proteins may be related to the regulation of AMPK, which needs further study.

Autophagy has been shown to affect inflammatory response, just as Kimura et al. reported that autophagy protected kidney from a variety of kidney inflammation, including acute, chronic, metabolic and aging-related inflammation (47). An experiment showed that enhanced autophagy can attenuate the inflammatory response mediated by NF-κB signaling pathway in macrophages, while the inhibition of autophagy can lead to inflammatory response (48). Emerging evidence have shown that NF-κB-mediated inflammatory response and its downstream factors, including inflammatory cytokines, are important factors in mediating the pathogenesis of NAFLD (49, 50). In the presence of lipotoxicity, IR and inflammation promote each other, forming a vicious cycle that further accelerates the development of NAFLD. ERα has many physiological functions, its activation is before NF-κB signaling pathway, and the deficiency of ERα is associated with hepatic lipogenesis (51, 52). In our study, ERα protein levels were reduced and NF-κB-mediated inflammatory response were activated, which further supported the occurrence of NAFLD induced by perinatal exposure to BPA in offspring.

Limitations of this study are that almost all of the data are only correlation studies, and no further study on the mechanism of BPA action through gene agonists or inhibitors. In addition, the number of pregnant rats *n* = 6/group was relatively small.

## Conclusion

In conclusion, perinatal exposure to BPA causes the development of NAFLD in both female and male offspring, which is associated with up-regulation of lipogenic genes, dysregulated autophagy, and activated inflammatory response involving PI3K/Akt/mTOR and TLR4/NF-κB pathways. BPA can affect lipid metabolism through multiple signaling pathways in liver, leading to the development of NAFLD.

## Data Availability

All datasets generated for this study are included in the manuscript/supplementary files.

## Ethics Statement

The animal study was reviewed and approved by the Animal Use and Care Committee at China Medical University. Written informed consent was obtained from the owners for the participation of their animals in this study.

## Author Contributions

L-HJ contributed conception and design of the study. RL, DW, F-JW, YM, J-HZ, and X-GW performed the experiments. RL and DW analyzed the data. L-HJ and RL wrote the manuscript. All authors contributed to manuscript revision, read and approved the submitted version.

### Conflict of Interest Statement

The authors declare that the research was conducted in the absence of any commercial or financial relationships that could be construed as a potential conflict of interest.
